# Primary cutaneous eccrine carcinoma in an elderly female with *PALB2* deletion

**DOI:** 10.1097/JW9.0000000000000289

**Published:** 2026-07-23

**Authors:** Seda Grabauskas, Patrick McMullan, Marti Rothe, Gillian Weston

**Affiliations:** a University of Connecticut School of Medicine, Farmington, Connecticut; b Department of Dermatology, University of Connecticut Health Center, Farmington, Connecticut

**What is known about this subject in regard to women and their families?**

- With a global rise in breast cancer incidence, breast carcinomas have become an increasingly significant health concern among women.
- In women with prior breast carcinoma, it is important to distinguish primary skin malignancies from metastatic disease.
- Primary cutaneous eccrine carcinoma diagnosis can be challenging, particularly within patients with a prior history of extracutaneous primary or metastatic adenocarcinoma.

**What is new from this article as messages for women and their families?**

- To our knowledge, this is the first report of primary cutaneous eccrine carcinoma in a female with a PALB2 mutation and prior bilateral invasive ductal carcinoma.
- This case highlights the importance of considering primary cutaneous eccrine carcinoma in patients with previous breast carcinoma.
- Our case letter underscores the need for awareness of potential associations between genetic mutations and rare skin malignancies.
- In patients with known visceral cancers, a comprehensive workup of immunohistochemistry, imaging, and collaboration among specialties is warranted.

## Introduction

Primary cutaneous eccrine carcinoma (PCEC) is a rare sweat gland tumor with nonspecific clinical features and heterogeneous histologic patterns, often mimicking those of primary breast adenocarcinomas or metastases.^1^ Accurate diagnosis is challenging, particularly in patients with a prior history of extracutaneous adenocarcinoma, requiring a thorough, multidisciplinary workup. We report a case of PCEC in a female patient with a history of bilateral triple-negative lobular breast cancer and a partner and localizer of BRCA2 (*PALB2*) mutation.

## Case report

**Fig. 1.**
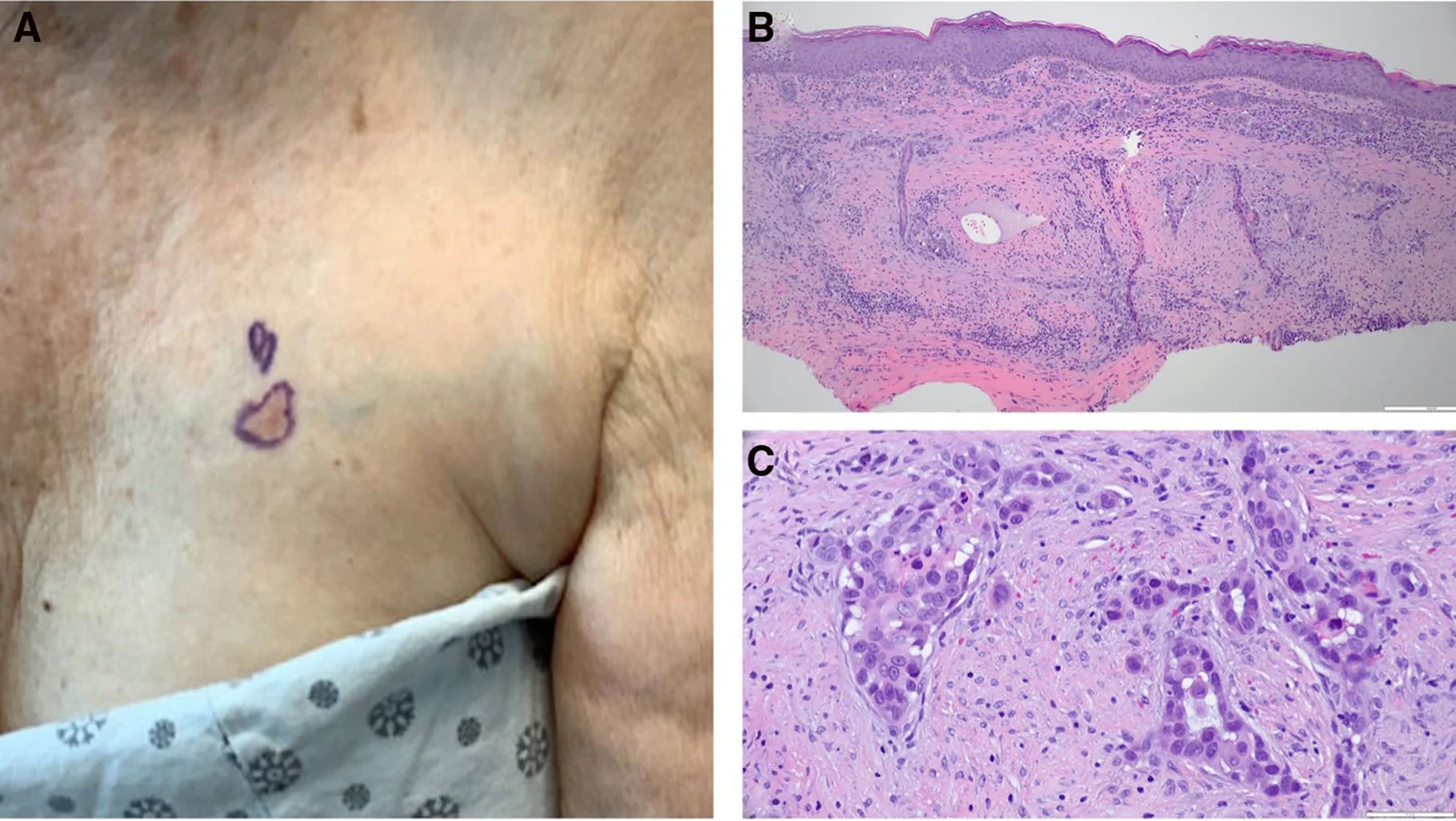
(A) Initial presentation of an asymptomatic 8 × 5 mm pink papule on the left anterior chest. (B) Routine staining of this shave biopsy demonstrates irregular cords, strands, and aggregates of atypical cells set within a scar-like stroma (100×). (C) Aggregates of atypical cells are characterized by duct-like spaces, nuclear pleomorphism, and single-cell necrosis (400×).

A 77-year-old woman with a 10-year history of ductal carcinoma in situ and bilateral triple-negative lobular carcinoma (treated with bilateral mastectomy, taxol-carboplatin chemotherapy, and anastrozole) under active surveillance because of genetic testing revealing a *PALB2* deletion (*PALB2* c.172_175del) mutation presented to a dermatology clinic for a full skin examination. Her cutaneous exam identified an 8 × 5 mm nonpruritic, nonpainful pink papule on her left anterior chest without palpable lymphadenopathy (Fig. 1A).

Shave biopsy revealed atypical glandular cells forming clusters, cords, and occasional glands within a scar-like stroma (Fig. 1B) and, on higher magnification, revealed moderate nuclear pleomorphism, increased mitoses, scattered squamous differentiation (including dense cytoplasm and intercellular bridges), and no lymphovascular invasion (Fig. 1C). Given these features, the differential diagnoses included PCEC, primary breast adenocarcinoma, or metastatic visceral adenocarcinoma.

**Fig. 2.**
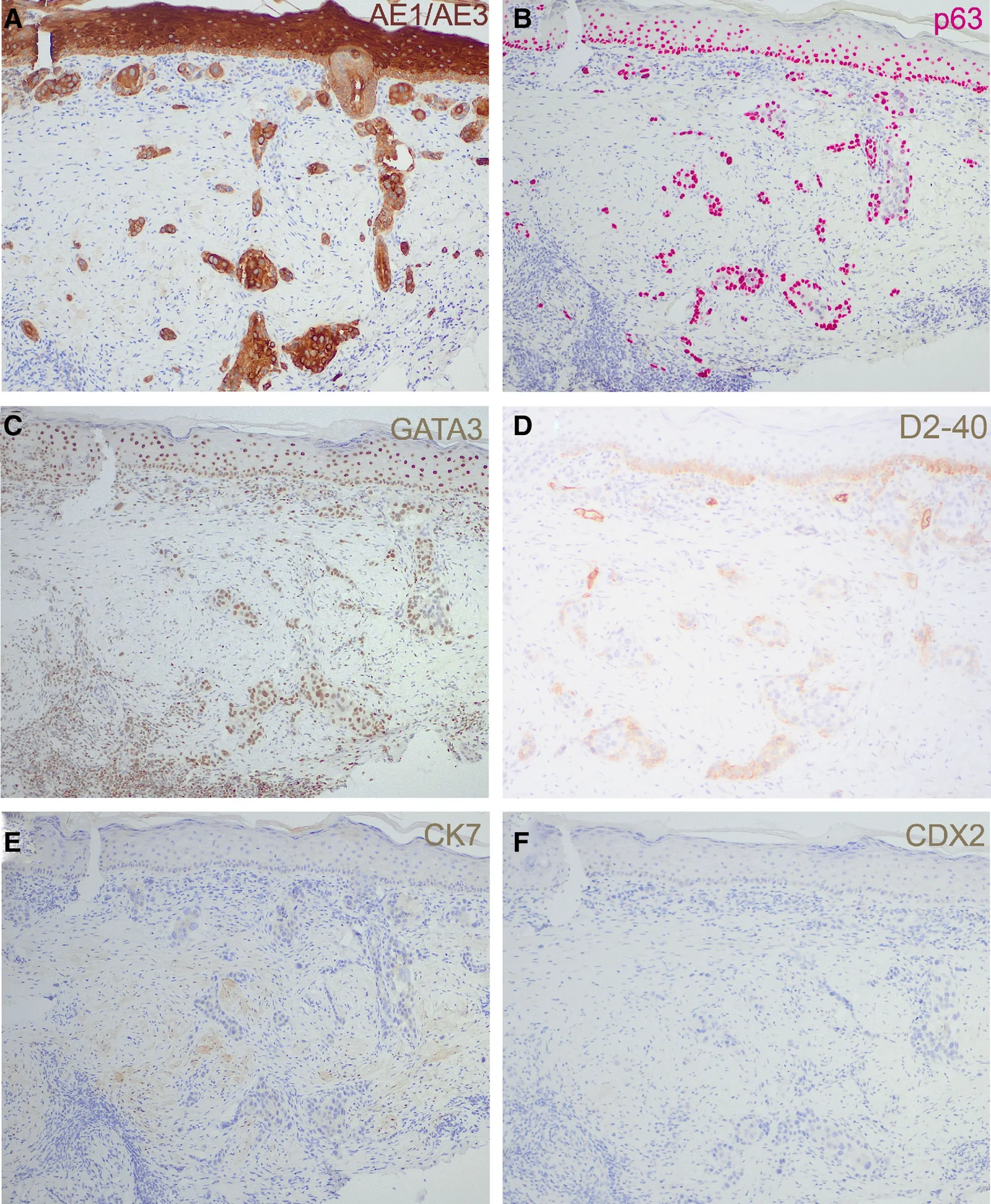
An immunohistochemical panel was performed and demonstrated the following profile in the cells of interest: (A) AE1/AE3 positive, (B) p63 positive, (C) GATA3 positive, (D) patchy and focal expression D2-40, (E) CK7 negative, and (F) CDX2 negative.

Immunohistochemistry (IHC) showed diffuse positivity for AE1/AE3, p63, GATA3, EMA, and ER; patchy D2-40 and focal carcinoembryonic antigen expression; and negative CK7, CK20, PAX8, and CDX2 (Fig. 2). Comparing this lesion to the patient’s previous breast adenocarcinoma highlighted distinct histologic and immunophenotypic features, notably squamous differentiation. Positron emission tomography–computed tomography scans showed no distant malignancy. These findings supported a diagnosis of PCEC, for which the patient underwent wide local surgical excision with no evidence of recurrence.

## Discussion

We describe, to our knowledge, the first report of PCEC in a female with a *PALB2* mutation and prior bilateral invasive ductal carcinoma. *PALB2* encodes a tumor suppressor involved in homologous recombination repair; mutations increase risks for breast, ovarian, and pancreatic cancers.^1^ Its role in cutaneous malignancies remains unclear, although prior cases of *PALB2*-associated cutaneous adnexal carcinomas have been documented in males without personal cancer history,^2,3^ suggesting that PCEC may be within the expanding PALB2-related tumor spectrum.

PCEC diagnosis can be exceedingly challenging, particularly in patients with a prior history of extracutaneous primary or metastatic adenocarcinoma, as these entities all share similar histologic features on hematoxylin and eosin staining.^4^ In this case, the poorly differentiated glandular and squamous atypical aggregates raised suspicion; however, IHC results helped delineate the diagnosis. Positivity for p63 and D2-40, along with negative CK7 and CK20, pointed toward a primary cutaneous origin, aided further by the absence of evidence for metastatic disease on imaging.^5^ This highlights the importance of comprehensive IHC panels –including cytokeratins, p63, and D2-40 – to differentiate primary skin tumors from metastases, especially in patients with prior cancers.

Our case underscores the need for awareness of potential associations between genetic mutations and rare skin malignancies. In patients with a history of visceral malignancies, diligent diagnostic workup – including IHC, imaging, and collaboration – is essential to accurately differentiate primary cutaneous neoplasms from metastases.

## Conflicts of interest

None.

## Funding

None.

## Study approval

This case report did not require Institutional Review Board approval according to institutional policy.

## Author contributions

SG: Conducted the literature review and drafted the manuscript. PM: Contributed to patient care, clinical data acquisition, the literature review, and manuscript revision. GW: Interpreted the histopathologic and immunohistochemical findings and revised the manuscript. MR: Supervised patient care and revised the manuscript. All authors approved the final manuscript.

## Patient consent

Patient has provided informed consent for publication of the case.
